# Supplementary figures and images for: Proteomic characterization of GSK3β knockout shows altered cell adhesion and metabolic pathway utilisation in colorectal cancer cells
Source: PLoS One. 2021 Nov 5;16(11):e0246707. doi: 10.1371/journal.pone.0246707 (PMC8570494; doi:10.1371/journal.pone.0246707)

Fig 2D

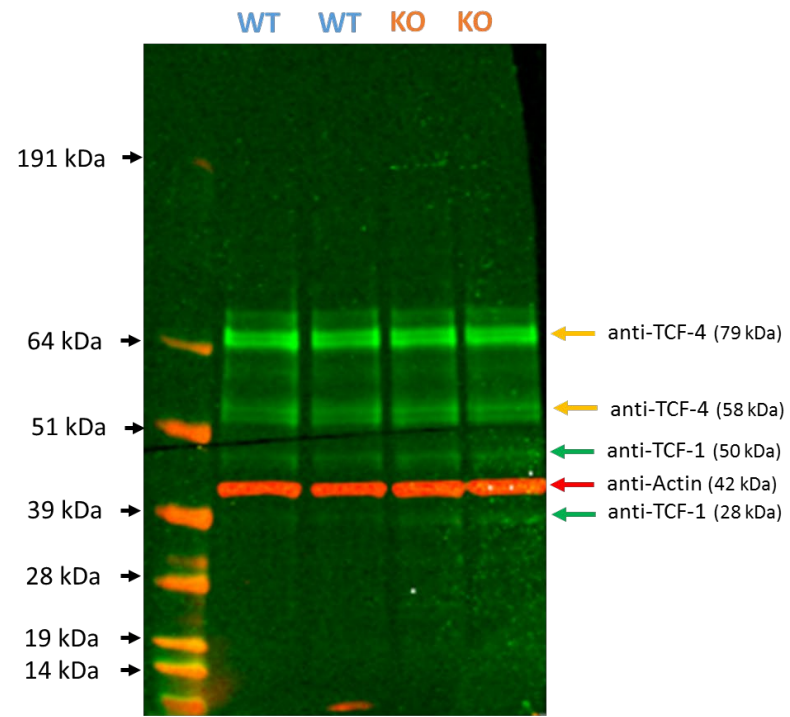

Fig 2D

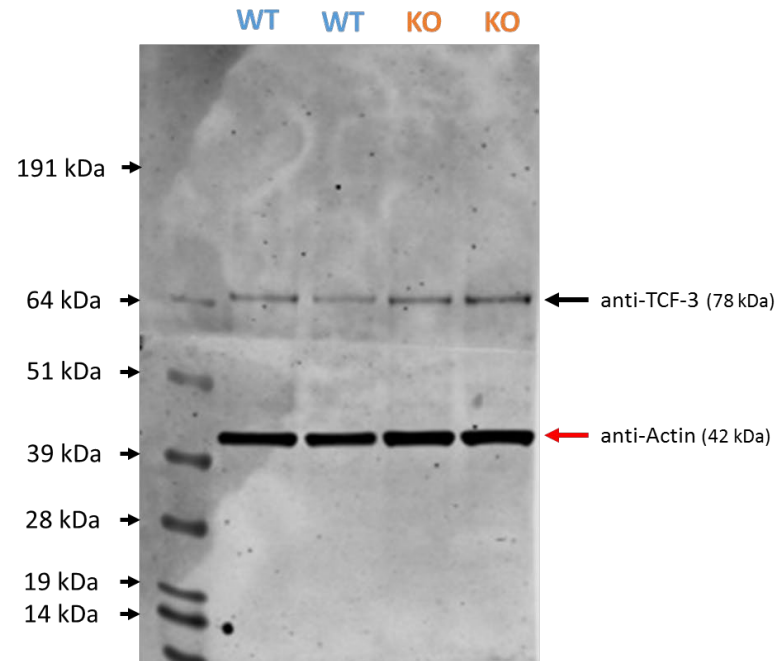

Fig 2E

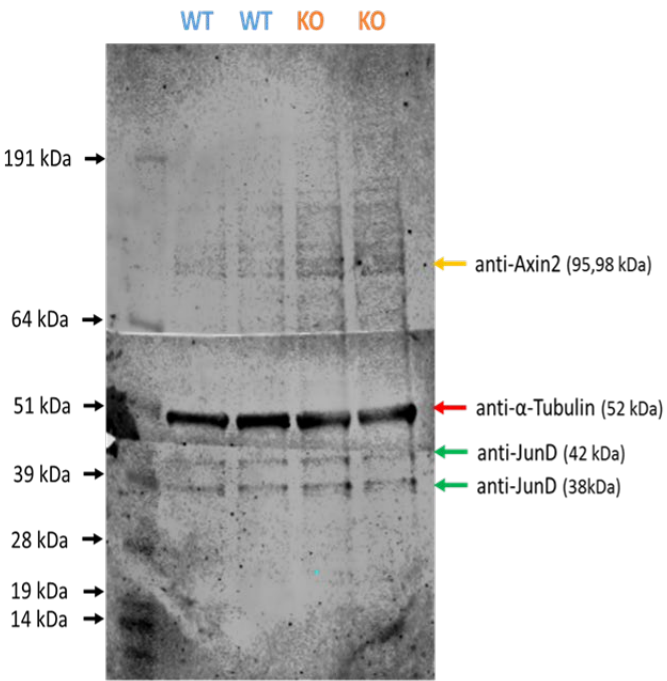

Fig 2C

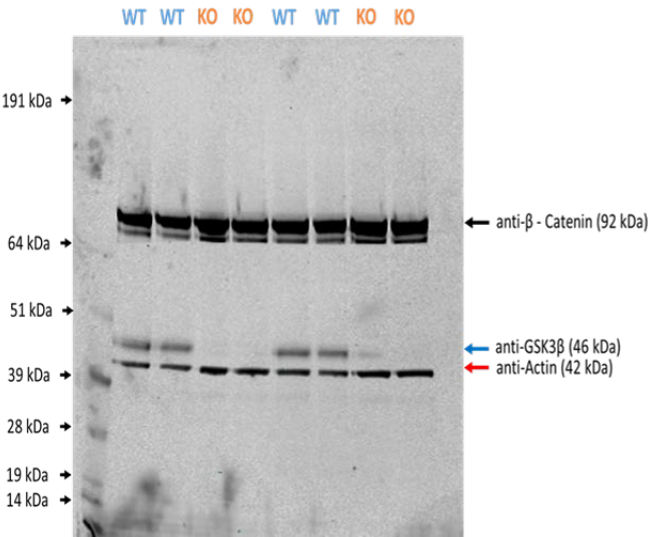

Fig 2B

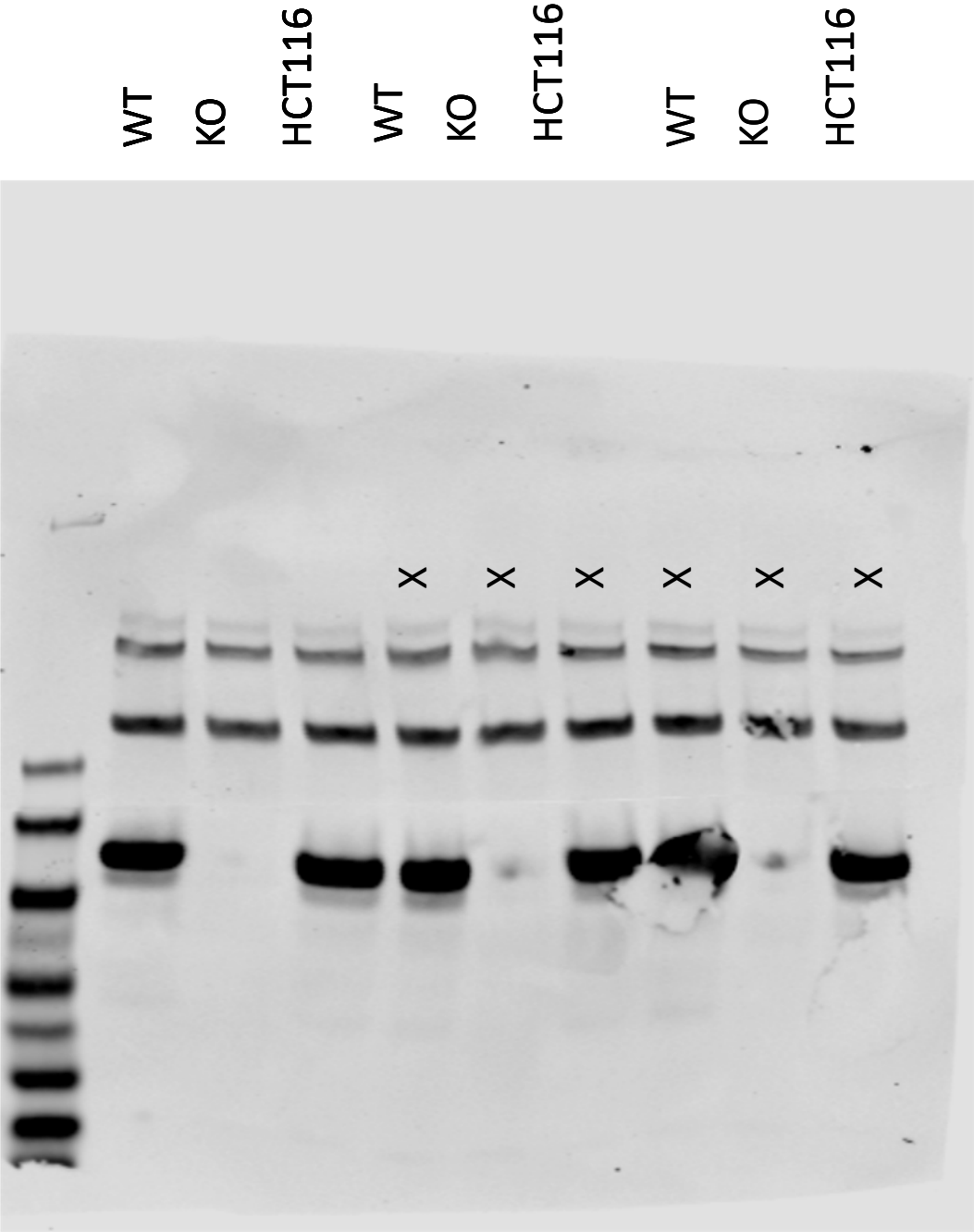

Same blot  
(with Actin)

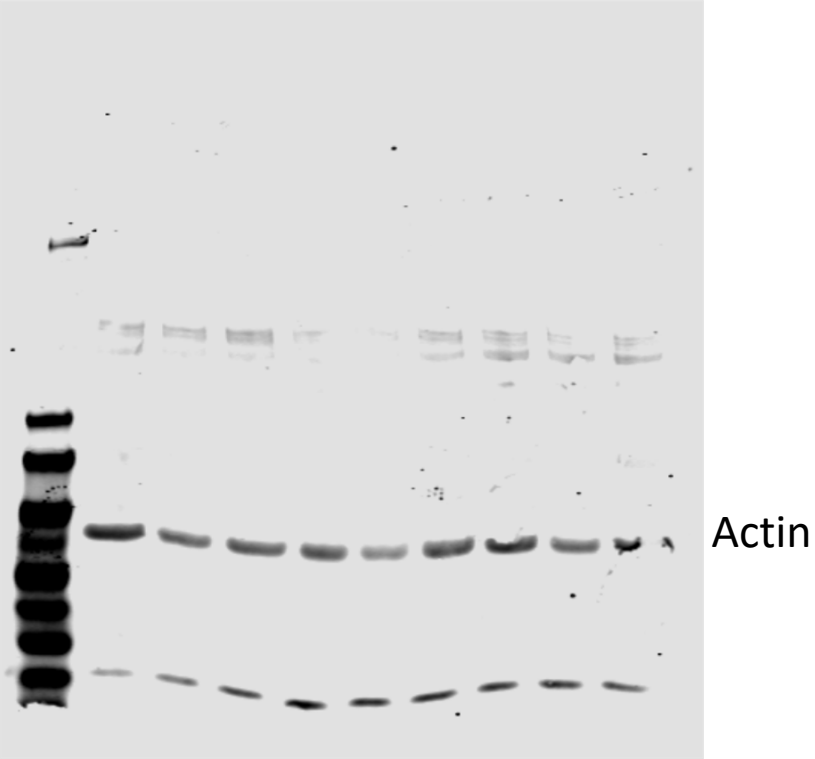

Fig 3B

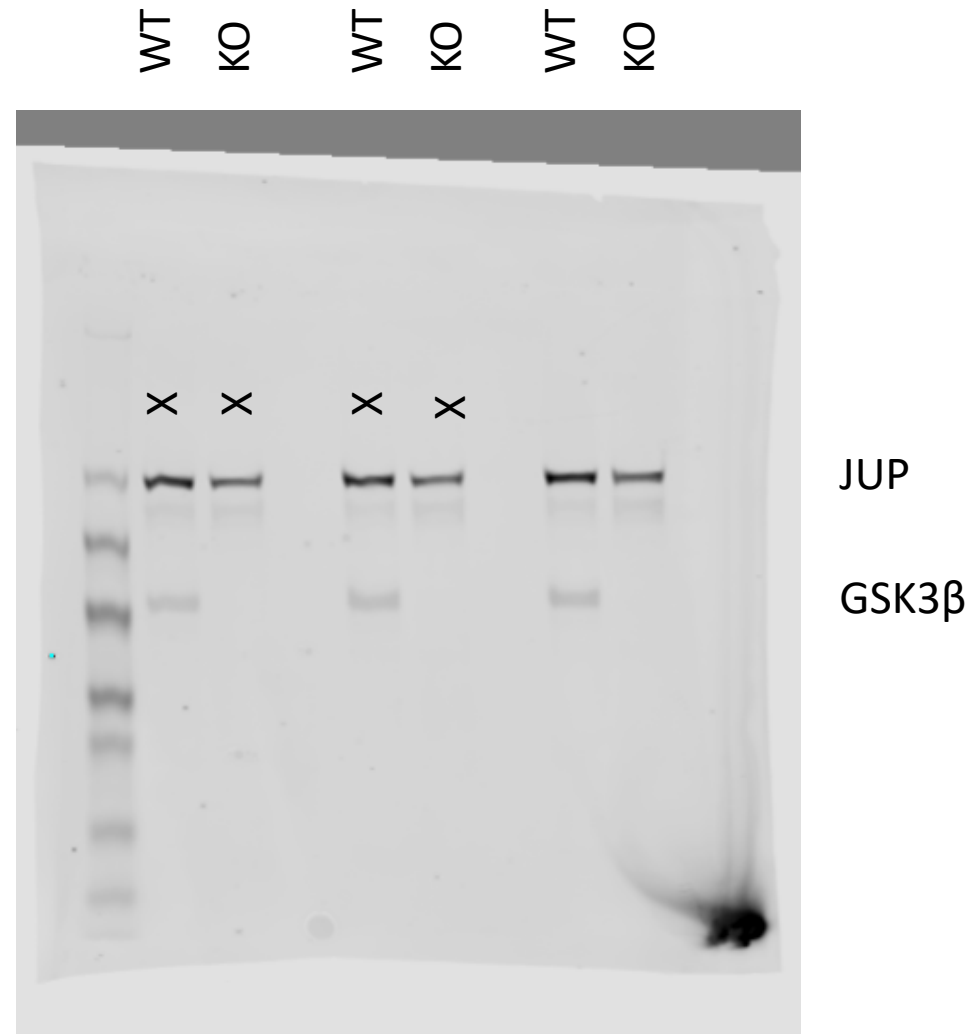

# Fig 3C

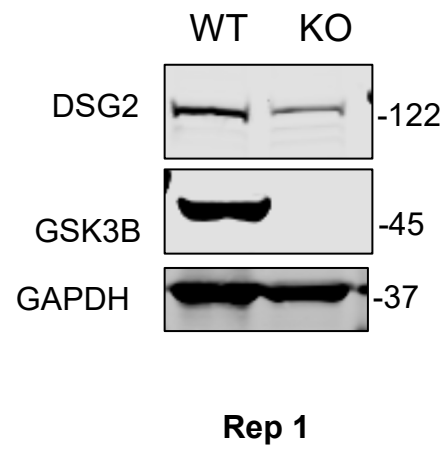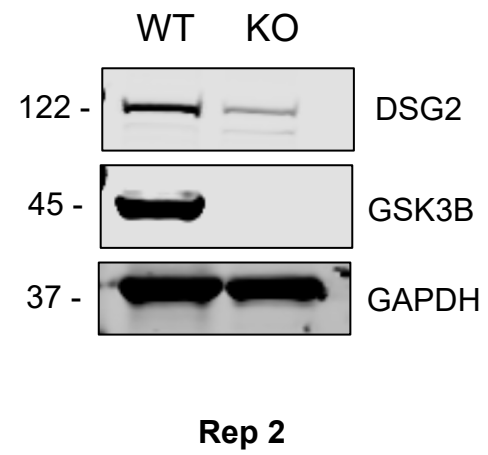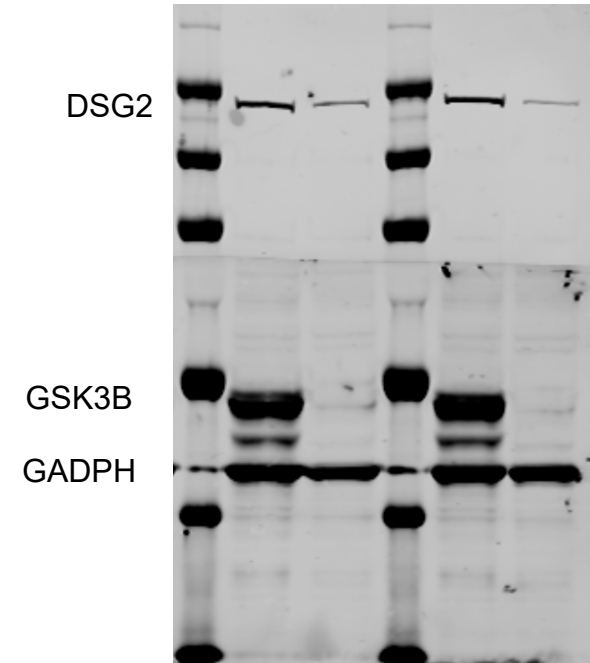

Fig 3D

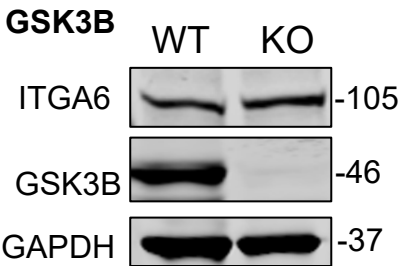

Rep 1

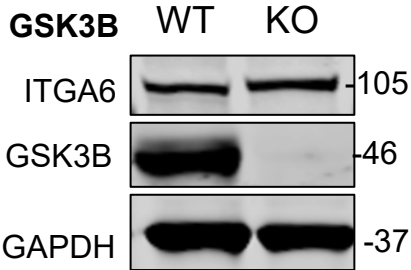

Rep 2

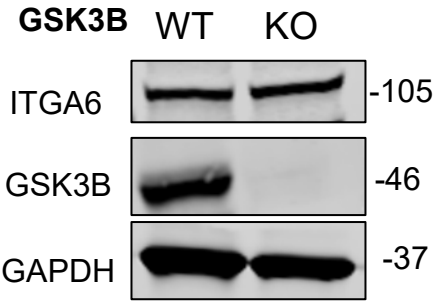

Rep 3

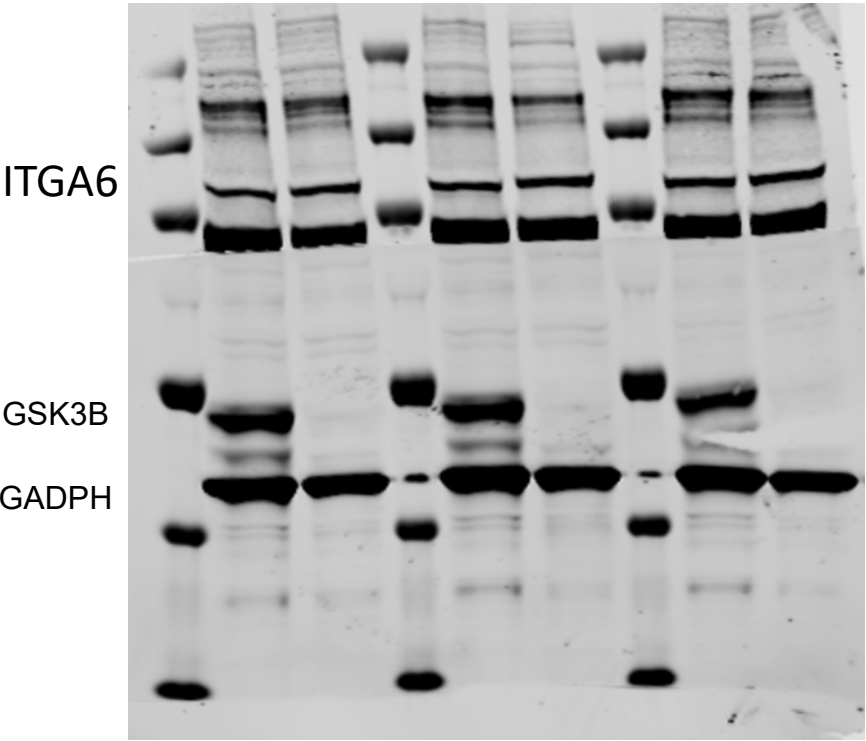

Supplement: S1 Raw images — (PDF) [file pone.0246707.s001.pdf]
