# Supplementary material for: Proteomic characterization of GSK3β knockout shows altered cell adhesion and metabolic pathway utilisation in colorectal cancer cells
Source: PLoS One. 2021 Nov 5;16(11):e0246707. doi: 10.1371/journal.pone.0246707 (PMC8570494; doi:10.1371/journal.pone.0246707)
Supplement: Attachment — Submitted filename: comments.docx [file pone.0246707.s004.docx]

**Page 3, Abstract**

- *Consistent with the role GSK3β, we found that… ->* Consistent with the role of GSK3β, we found that….
- *…therefore use this knockout cell model ->…* therefore used this knockout cell model
- *…and we characterize the phenotype and altered proteomic profiles associated with this -> ….*and we characterized the phenotype and altered proteomic profiles associated with this
- *We also characterize the perturbation…->* We also characterized the perturbation…
- *…and identify defects*  *->* and identified defects

**Materials and methods**

- Several times the 6-well plates have been indicated as 6-cell plates

**Pag.12**

- *We tested the canonical Wnt-driven transcriptional activity of these cells through reporter (TopFLASH) analysis as shown in Figure 1G.*

It’s Figure 2G

- *Finally, we identified additional Wnt signaling components in the mass-spectrometry data. β-catenin showed no significant difference between WT and KO cells, whilst APC (Adenomatous Polyposis Coli), an important component of the destruction complex was only identified in WT cells, indicating that loss of GSK3β may result in loss or reduced expression of APC. Finally, the Casein Kinase 1 alpha subunit (CSNK1A1) which phosphorylates β-catenin at S45 showed reduced expression in knockout cells (p-value = 0.013).*

The Authors show the western blot for all those proteins whose expression does not change in the absence of GSK3B but do not show the western blot for the only proteins whose expression is indeed changed: It’s a non-sense! So please add the blots for APC and CSNK1A1

**Pag.14**

- *The HCT116- GSK3β-KO cells showed a specific reduction in protein abundance of desmoplakin (DSP), plakoglobin (JUP) and desmoglein (DSG), and an increase in abundance of their essential linker proteins, plakophilins.*

*We also observed the complete lack of expression in HCT116-GSK3β-KO cells of two key integrin family members (Figure 3A). ITGA6 and ITGB4*

In the western blot of Fig.3 (that should be presented as a separate panel, 3B) not all of the proteins identified ad differentially expressed in KO cells by MS are validated and in the text is mentioned a protein, desmoglein, whose expression is not presented in the figure neither as MS nor as WB data. Please show the expression of all the proteins both by MS and WB. In addition, remember that there are rules as of proteins have to be indicated: full names followed by acronyms in brackets should be indicated in the text the first time a protein is mentioned and acronyms can be used in the figures but they have to explained in the legend. So please correct the text and the legend accordingly. In fact, for desmoplakin and plakoglobin the acronym is indicated in the text but for plakophilins it is not. Then the Authors report of the complete loss of expression *of two key integrin family members* but they don’t specify who they are….???? It’s up to the reader to guess they are ITGA6 and ITGB4 whose function is explained in the following sentence. By the way, these two proteins are indicated by acronyms without displaying the full name…. Finally, in figure 3 proteins are indicated by their full name for MS experiments and by acronyms in the WB panel!!... that are not explained in the figure legend… this is extremely confusing for the reader!

- *Following mechanical agitation of detached monolayers of HCT116-*

*GSK3β-KO cells the cell monolayer fragmented into numerous pieces, in contrast to the HCT116-GSK3β-WT cells in which the monolayer remained intact*

Is it possible a quantification of the phenomena for example, by counting the fragments in a given area?

**Pag.15**

- *a soft agar colony growth assay was performed and colony number over 6*

*days recorded (Fig 4C). Results indicate that loss of GSK3β did not incur any colony-forming advantage to HCT116-GSK3β-KO cells*

First of all, usually the results of a soft agar assay are shown as images of the cristal violet-stained welsl accompanied by the graph showing the quantification. Only the graph is not acceptable.

Second, the soft agar assay is an endpoint assay ie, colony forming in two different experimental situation are counted at the end of the experiment only, and not every day. This because, by definition, a colony is considered when it is formed by at least 50 cells: given that HCT116 have a cell cycle of approximately 24hs it means that a colony is formed between day 6 and day 7.

Therefore I request a “real”experiment of soft agar with representative images of the colonies after 7-10 days, accompanied by quantification graphically shown

Finally, what do you mean for:

- *although we observed an increased cell-count in cultures of HCT116-GSK3β-WT as compared to HCT116-GSK3β-KO (Figure 4C).*

Reading this sentence I understand that the Authors are referring to a different experiment like a growth curve (which is actually performed in cultures) not to a soft agar assay. Please explain

**Figure 5**

**Legend of panel C**

There’s no legend of the graph: please indicate what the colors and empty/filled squares means

**Growth curve shown in panel D**

From day 6 to day 9 a plateau for WT cells and a decrease in survival of KO cells is evident, likely to be due to nutrients exhaustion. To assess the growth potential of a cell line cells should be plated at 10% and then counted every 24 hs in the log phase that in this experiment appears to be from day 1 to 6. Why the Authors decided to carry on for 3 more days? They exposed cells to an “artificial” stress in the final part of the experiment, so the apparent reduced “growth potential” (as they indicate it at page 17) of KO cells is in reality an a decline in the number of cells due to increased death of cells in conditions of nutrient exhaustion. WT cells seem instead to cope better in face of such a stress since the decrese in the number of cells is minimal. In order to get reliable and interpretable results the Authors should repeat the experiment in better controlled conditions such as stopping the growth count at 6 days. It is clear from the data they showed that WT cells are more resistant to limited nutrient availability so I would perform more experiments in that sense ie, repeating growth curves also maintaining cells in reduced amount of serum (eg in 1% serum) or assessing their viability in absence of serum by FACS analysis or by CellTiter-Glo® Luminescent Cell Viability Assay or even by a very simple Trypan blue staining.

**Pag.19**

- *Loss of DSP phosphorylation by knockdown or knockout of GSK3β results in the formation of desmosomes lacking a connection to the cytoskeletal intermediate filaments, reducing the strength of desmosome cell-cell junctions (24). GSK3 and PRMT1 have been shown to cooperatively catalyse posttranslational modification of desmoplakin and inhibition of GSK3 resulted in delayed formation of adherent junctions (8). Intriguingly, upon knockdown of GSK3β, DSP aggregates at interactin filaments in the cytoplasm which is mediated by GSK3β phosphorylation of DSP at S2849, without affecting the protein abundance of DSP (8). This relocalization of the protein results in a disconnection of the desmosomal plaque from the intracellular cytoskeleton, reducing the strength of desmosomal plaques. Relocalization of DSP may occur in the HCT116-GSK3β-KO cells as a result GSK3β loss, explaining the observations we have made*.

In this paragraph the Authors refer indiscriminately to GSK3 and GSK3*β* but this is not correct since the alpha and beta isoforms, although being largely redundant play also unique roles as demonstrated by gene knockout studies. In fact, GSK3A is unable to rescue the lethal phenotype of GSK3B null mice: the animals die during embryogenesis as a result of liver degeneration caused by widespread hepatocyte apoptosis, where excessive TNF-alpha-mediated cell death occurs, due to reduced

NFkB function (Hoeflich Ket al., 2000, Nature 406: 86–90). On the other hand, GSK3A null mice are viable and show metabolic defects – such as enhanced glucose and insulin sensitivity and reduced fat mass - which cannot be counteracted by the beta isofom (MacAulay K, et al.,2007, Cell Metab 6: 329–337). The reference used here to support their reasoning is vague about which isoform is capable to mediate the effects described since these Authors mostly use in their experiments LiCl which is a inhibitor of both isoforms.Therefore, it would be better to find an appropriate reference or modify the discussion.

**Pag.19**

- *These findings are in line with the extensive role that GSK3β plays in the regulation of metabolic pathways, including the early finding that glycogen synthase is a substrate of GSK3β kinase activity (25).*

Also in this case the statement is not well supported by the citation quoted. In fact, the reference is the original paper describing the glycogen synthase as a substrate of GSK3β. What about papers supporting *the extensive role that GSK3β plays in the regulation of metabolic pathways?*  To my knowledge is more GSK3A than GSK3B that plays a role in the metabolis, given that GSK3A null mice are viable and show metabolic defects which cannot be counteracted by the beta isofom (see above). Therefore please quote the data from the literature supporting this statement or modify the discussion. Moreover, given that the data from Authors indicate a role for GSK3B in metabolic regulation can they exclude that the knockout of the beta isoform do not trigger a compensatory upregulation of GSK3A?
